# Table to text generation with accurate content copying

**DOI:** 10.1038/s41598-021-00813-6

**Published:** 2021-11-23

**Authors:** Yang Yang, Juan Cao, Yujun Wen, Pengzhou Zhang

**Affiliations:** State Key Laboratory of Media Convergence and Communication, Communication University of China, Beijing, 100024 China

**Keywords:** Engineering, Mechanical engineering

## Abstract

Generating fluent, coherent, and informative text from structured data is called table-to-text generation. Copying words from the table is a common method to solve the “out-of-vocabulary” problem, but it’s difficult to achieve accurate copying. In order to overcome this problem, we invent an auto-regressive framework based on the transformer that combines a copying mechanism and language modeling to generate target texts. Firstly, to make the model better learn the semantic relevance between table and text, we apply a word transformation method, which incorporates the field and position information into the target text to acquire the position of where to copy. Then we propose two auxiliary learning objectives, namely table-text constraint loss and copy loss. Table-text constraint loss is used to effectively model table inputs, whereas copy loss is exploited to precisely copy word fragments from a table. Furthermore, we improve the text search strategy to reduce the probability of generating incoherent and repetitive sentences. The model is verified by experiments on two datasets and better results are obtained than the baseline model. On WIKIBIO, the result is improved from 45.47 to 46.87 on BLEU and from 41.54 to 42.28 on ROUGE. On ROTOWIRE, the result is increased by 4.29% on CO metric, and 1.93 points higher on BLEU.

## Introduction

Natural language generation based on tabular data, also known as table-to-text generation, takes tabular data as input and generates human-like expressions of text. It has been applied in various scenarios, such as biography generation, NBA game generation, and weather forecasting^1–3^. In general, table-to-text generation is divided into two subtasks: content selection and surface realization. Content selection mainly determines what content to select from the input table, whereas surface realization primarily generates text from the selected content. In recent years, algorithms based on neural networks have been developed that no longer solve these two subtasks separately, and they have achieved remarkable results in different domains^4–6^.

Most existing table-to-text generation approaches are based on encoder-decoder frameworks, which are RNN-based models^7,8^. Although significant progress has been made, we believe that there are four key problems. Firstly, there are a lot of attribute information and noise data in the table, but they rarely appear in the text. Although the RNN-based models can obtain position information, they are unable to capture the long-term relationships between the table and the text. Therefore, a model is needed to handle the correlation between the table and the text. Secondly, tables for different application scenarios, such as the ROTOWIRE dataset for NBA game generation^9^ and the WIKIBIO dataset for biography generation^4^, have different structures and types. For the ROTOWIRE dataset, there are 23 and 15 different types of values described in box-score tables and line-score tables, respectively, with a vocabulary size of 11.3 K words. Compared to the ROTOWIRE dataset, WIKIBIO includes more types of attribute information, with an average of 20 attributes per table, and a larger vocabulary of approximately 400 K words. So, a good algorithm is needed to express tabular data of different structures and types and obtain an accurate semantic representation of the source table. In addition, some proper nouns, such as names and places, often appear in tabular data, and they rarely appear in vocabulary; therefore, these nouns are called “out-of-vocabulary”. Using the copy mechanism^10^ to copy words from a table to replace unknown words in the output text can lead to problems regarding inaccurate copying. Finally, at the text generation stage, a maximization-based search strategy, such as beam search, is used to select tokens with high probabilities as outputs, leading to text degradation. Humans would give human-like grammatical text a higher probability of being output than well-formed text, and problems such as repetitive and incoherent text occur.

In this paper, we aim to solve the above challenges. We propose a novel table-to-text generation framework and introduce two auxiliary learning tasks. Our contributions are as follows:

1. We propose a novel transformer-based model to process various data-to-text generation tasks.
2. we utilize multi-task learning with two auxiliary tasks, table-text constraint loss and copy loss. In detail, the table-text constraint loss task is introduced to process complex tabular data with different structures and types. Besides, we add a copy loss task to exactly guide model.
3. We change the search strategy for generated text to reduce the probability of generating repetitive text.

Experiments are conducted on the WIKIBIO dataset and ROTOWIRE dataset to demonstrate the effectiveness and generality of our model.

## Related work

Table-to-text generation has attracted widespread attention, aiming to help humans better understand tabular data. We classify table-to-text generation into two groups: pipeline pattern and end-to-end methods. Early data-to-text generation methods follow the pipeline pattern that divides generation into content selection and surface realization. Pipeline pattern relies heavily on rule-based and template-based approaches, which typically involves selecting the correct rule set or retrieving the appropriate template for the generation task at hand^13,14^.

In recent years, due to the emergence of massively parallel datasets such as WIKIBIO, end-to-end neural network methods have become a research focus. In^15^ proposed the neural checklist model to address the problem of repeated information generation in structured data for recurrent neural network (RNN) models. The model is applied to the generation of menus, where dish names and ingredient lists are the inputs, and the machine outputs the corresponding recipes. Text generation based on structured data suffers from data sparsity, and many attributes and values in structured data rarely occur, making it challenging for the algorithm to learn the model. In^4^ introduced the copy mechanism into the neural language model to cope with the problem of sparse data. Based on the conditional neural language model, the structured data are parsed locally and globally, with a focus on the attribute information in the data. In^16^ introduced multiple decoders with hidden variable factors to specify which decoder generated the final text based on the classical sequence-to-sequence model. Learning is enhanced by setting up multiple submodels that are only responsible for processing specific data expressions. In^17^ explicitly modeled content selection and content planning in an end-to-end neural network architecture. The generation task is divided into two stages by first conducting content selection and content planning operations to highlight the content and order of information that should be mentioned and then taking the generated content plan as input and outputting the text. Additionally, to increase the interpretability and controllability of the models, a number of models have recently emerged that combine end-to-end approaches with traditional rule-based and template-based approaches. In^18^ used a hidden semi-Markov model (HSMM) to model text and parameterized all probability terms with a neural network. After completing the training of the model, the Viterbi algorithm is used to obtain templates for text generation.

Although the above algorithms have achieved promising results, the use of RNN-based models fails to capture long-term dependencies. In^19^ used the transformer-based model for machine translation. The sentence-level agreement module is used to minimize the differences between the source and target sentences, resulting in a close distribution of sentence-level vectors between the source and target sides. In^20^ presented a transformer-based data-to-text generation model that learns content selection and surface realization in an end-to-end manner. It improves the correctness of the output by modifying the input representation; it also adds an additional learning objective for content selection modeling and achieves good results on game summaries. In^21^ proposed a few-shot table-to-text generation. Model uses a powerful pre-training model (GPT-2) and two auxiliary learning tasks, outperforming state-of-the-art baselines on three few-shot datasets. A template-based table transformation module is employed to convert the table into a sequence. Two auxiliary learning tasks of table structure reconstruction and content matching are used to solve the pre-training model’s lack of table structure modeling and text fidelity. In^22^ proposed a general knowledge-based pre-training model (KGPT) to deal with various text generation tasks, and achieved powerful performance with few samples and zero samples. They first pre-train the model on the constructed knowledge-based KGTEXT dataset, and then fine-tune the model on downstream tasks like WikiBio^4^, WebNLG^23^ and E2ENLG^24^. In^25^ proposed a new algorithm to solve the problem of faithful table-to-text generation. Two faithful generation methods are proposed: generation according to the augmented plan and selection of training examples based on faithfulness ranking. In addition, two new metrics are introduced to evaluate generation faithfulness. In^26^ proposed an end-to-end model to generate entity descriptions. They adopt the joint learning of text generating and content-planning to deal with disordered input, and apply the content-plan-based bag of tokens attention mechanism to highlight salient attributes in an appropriate order.

## Table-to-text generation

The task of table-to-text generation is to take a structured table, $T=\{(f_{1},v_{1}),(f_{2},v_{2}),...,(f_{m},v_{m})\}$ as input and output a natural language description that consists of a sequence of words $y=\{y_{1},y_{2},...,y_{n}\}$ . Each input sentence $T_{i}=\{f_{i},v_{i}\}$ consists of a field $f_{i}$ and its corresponding sequence of word fragments $v_{i}=\{w_{i}^{1},w_{i}^{2},...,w_{i}^{l}\}$. Here, *m* is the number of fields and values, *n* is the number of words in each description, and *l* is the number of words in each value.

**Figure 1:**
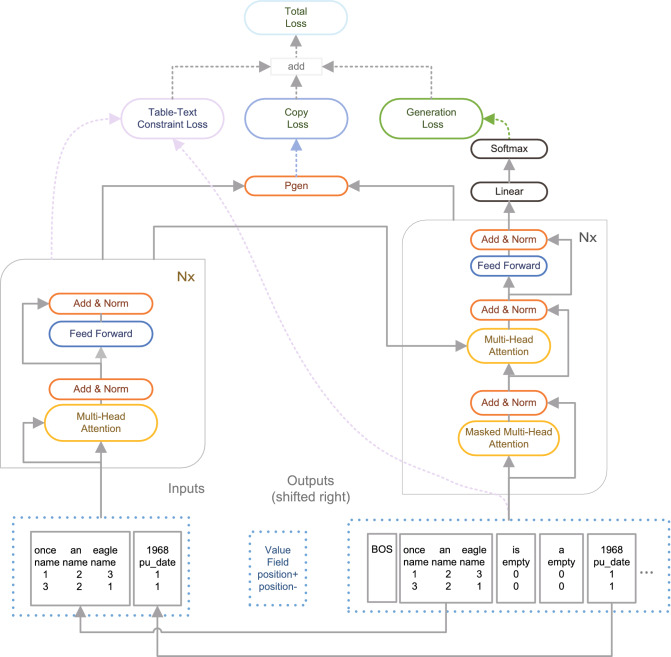
The framework of our model for table-to-text generation.

Figure 1 illustrates the overall framework of our model. Our model uses the encoder-decoder architecture. The encoder is composed of an input layer and N identical layers. Each layer has two sub-layers. The decoder consists of an input layer, N identical layers, a linear layer and a softmax layer. ”Nx” means a stack of N identical layers. In the experiment, we set N to 6. In ”Nx”, in addition to the two sub-layers of the encoder layer, the decoder layer adds a third sub-layer. The final output of the decoding is the probability distribution of the word at the corresponding position.

The model is designed from two aspects: table content copying and language modeling to generate target texts. In the training process, we propose two auxiliary learning tasks: table-text constraint loss and copy loss in addition to the traditional generation task.

### Transformer model

We adopt the transformer model^27^ as our base model. The transformer is based solely on a self-attention mechanism, thereby removing the recurrence and convolution operations completely. The self-attention mechanism has two sublayers, the multi-head self-attention defined by Eqs. (1–3) and feedforward networks defined by Eq. (4). Our proposed transformer-based table-to-text generation model learns to estimate the conditional probability of a text sequence from a source table input, as shown in Eq. (5).

1 $$\begin{aligned} MultiHead(Q,K,V)= & {} Concat(head_{1},...head_{h})W^{O} \end{aligned}$$

2 $$\begin{aligned} head_{i}= & {} Attention(QW_i^Q,KW_i^K,VW_i^V) \end{aligned}$$

3 $$\begin{aligned} Attention(Q,K,V)= & {} softmax\left(\frac{QK^{T}}{\sqrt{d_{k}}}\right)V \end{aligned}$$

4 $$\begin{aligned} FFN(x)= & {} max(0,xW_{1}+b_{1})W_{2}+b_{2} \end{aligned}$$

5 $$\begin{aligned} P(y|T;\theta )= & {} \mathop {\Pi }\limits _{i=1}^{n}P(y_{i}|y_{<{i}},T;\theta ) \end{aligned}$$

where $y_{<i}$ denotes the decoded words from previous steps and $\theta$ is the learnable transformer parameter.

### Constraint on source table and target text

#### Table representation

A table can be viewed as a record of multiple sets of field-value, where the values are word fragments corresponding to the fields. The structural representation of a table consists of field embeddings and context embeddings. We follow previous work^1^ and define a field embedding ${\hat{Z}}_{enc}=\{f_{enc};p_{enc}^{+};p_{enc}^{-}\}$ ,where $(p_{enc}^{+},p_{enc}^{-})$ includes the positions of the token counted from the beginning and the end of the field as the positional embedding of the token, replacing positional encoding in the transformer model. Therefore, the field embedding ${\hat{Z}}_{enc}$ and context embedding ${\hat{C}}_{enc}$ are concatenated to obtain the embedding representation of table ${\hat{X}}=\{ {\hat{Z}}_{enc};{\hat{C}}_{enc}\}$. We define $R_{enc}$ as a table representation of ${\hat{X}}$ via the self-attention layers in the encoder. $E_{dec}$ is the target text representation of *y* obtained by embedding layers in the decoder.

#### Table-text constraint loss

In many table-to-text datasets, tables have a large number of attributes and much noise data, and complex-structured table representations cannot be accurately obtained using a simple encoder. Target text has similar meanings to the source table, and therefore, it is possible to use target text to constrain complex-structured tables. We are inspired by machine translation^19^ to strengthen the source representation using a table-text constraint loss. Our table-text constraint loss $L_{CL}$ for measuring the distance between the table and target text is defined as:

6 $$\begin{aligned} L_{CL}=\Arrowvert {\hat{R}}_{enc}-{\hat{E}}_{dec}\Arrowvert ^{2} \end{aligned}$$

where ${\hat{R}}_{enc}=Mean(R_{enc})$ and ${\hat{E}}_{enc}=Mean(E_{dec})$ are the mean value embeddings of the source and target sentences, respectively.

### Pointer-generator network with copy loss

In this part, we use word transformation module and copy loss to guide the pointer-generation network^10^ correctly copy the table content.

#### Pointer-generator network

The pointer-generator network combines the seq2seq model with a pointer network, which maintains $p^{copy}$ to choose between copying from an input table or generating from a fixed vocabulary list. Therefore, the final word probability distribution is

7 $$\begin{aligned} p(y_{i})=(1-p^{copy})p_{vocab}(y_{i})+p^{copy}\sum _{i}a_{i}^t \end{aligned}$$

**Table 1:** Target text transformation results (bottom) based on Tabular data (top).

Field	Value
Name	Once an eagle
Author	Auton,myrer
Country	United States
Language	English
Genre	War
Publication_date	1968
.	.
Target text	Once an eagle is a 1968 war novel by American .
Transformation results	name_1_3 name_2_2 name_3_1 empty_0 empty_0 publication_date_1_1 genre_1_1 empty_0 empty_0 country_0 .

8 $$\begin{aligned} p^{copy}=sigmoid(W_{h}h_{t}+W_{s}s_{t}+W_{x}y_{t}^{new} +b) \end{aligned}$$

where $W_{h}$ ,$W_{s}$ , $W_{x}$ and *b* are learnable parameters; $p_{vocab}$ denotes the probabilities of generating the next word, $h_{t}=\sum _{i}a_{i}^th_{i}$ , $h_{i}$ are the hidden states of the encoder; and $y_{t}^{new}$, $s_{t}$ , and $a_{i}^t$ are the input of the decoder, the hidden state and the attention weights returned from the encoder-decoder attention module, respectively.

#### Copy loss

To provide accurate guidance to the pointer-generator network, we employ word transformation methods and auxiliary learning tasks in the model.

We first use word transformation methods to process the target text. When matching the words in the target text with the values in the table, if the word $y_{i}$ in the target text appears in the table, $y_{i}$ is replaced with the field and position information of the value in the table, such as (name,position+,position-), where “position+” and “position-” indicate the positions of the token counted from the beginning and the end of the field, respectively. Words that do not appear in the table are replaced by the word ”empty”, and the position information is recorded as zero. Table 1 describes the target text transformation results. For example, when the word ”war” of the target text appears in the table, we replace the word ”war” with (genre, 1, 1).

Additionally, we find that the value corresponding to the “country” attribute often has different expressions as word aliases. If another name for the word in the target text appears in the table, it is processed as above to obtain the corresponding field and position information of the target text. As shown in Table 1 , ”United States” in the table and ”American” in the target text do not match, but they represent the same country, so the field of ”United States” is used instead of ”American” in the target text. Finally, we concatenate the content embedding ${\hat{y}}_{dec}$, field embedding $f_{dec}$ , and position embedding $(p_{dec}^{+},p_{dec}^{-})$ of the target text as inputs for the decoder.

9 $$\begin{aligned} y^{new}=\{{\hat{y}}_{dec};f_{dec};p_{dec}^{+};p_{dec}^{-}\} \end{aligned}$$

At the position of the matching words, we maximize the $p_{copy}$. Our loss function is as follows:

10 $$\begin{aligned} L_{copy}=\sum _{y_{i}\in V}1-p_{i}^{copy} \end{aligned}$$

where *V* represents the value in the table and $y_{i}$ represents the target text at the position *i*.

### Search strategy

The sentences generated in the decoder phase are repetitive, incoherent, and boring. Even with sufficient input from the state-of-the-art BERT^28^ and GPT^29^ language models, it is hard to generate high-quality texts. The main reason for this phenomenon is the use of maximization-based search strategies, such as the beam search. The beam search algorithm takes the top n (width of the beam search) tokens at a time from the vocabulary with the highest probability, repeats the process until a terminator is encountered, and finally outputs the top n sequences with the highest scores. The algorithm usually assigns a higher probability to well-formatted texts than to poorly-formatted texts, but in long texts, high probability outputs tend to yield generic and repetitive sequences.

To address this phenomenon, we use a combination of nucleus sampling (top-p sampling)^12^ and the top-k sampling strategy^11^ as our search strategy. By truncating the unreliable tails of probability distributions, sampling from tokens containing the vast majority of high-probability words enables the model to avoid the generation of very low-ranked words and allows for dynamic selection.

#### Top-k-top-p sampling

The top *k* words $V^{(k)}\in V$ with the highest probabilities are selected from the vocabulary *V* to avoid generating very low-ranked words. The word in the vocabulary $V^{(k)}$ whose sum of probabilities is greater than the threshold *p* is then selected, and the original distribution is rescaled to a new distribution from which the next word is sampled. The size of the sampling set is dynamically adjusted according to the shape of the probability distribution at each time step.

11 $$\begin{aligned} p^{'}= & {} \sum _{x\in V^{(k)}}p(x|x_{1:i-1})\ge p \end{aligned}$$

12 $$\begin{aligned} p^{'}(x|x_{1:i-1})= & {} {\left\{ \begin{array}{ll} \frac{p(x|x_{i-1})}{p^{'}}&{} if \ x \in V^{(K)} \\ 0&{} otherwise \end{array}\right. } \end{aligned}$$

### Loss function

Our objective function *L* consists of three parts: table-to-text constraint loss function $L_{CL}$ , table-to-text generation loss function $L_{GL}$ and copy loss $L_{copy}$:

13 $$\begin{aligned} L= L_{GL}+\lambda _{1} L_{CL}+\lambda _{2}L_{copy} \end{aligned}$$

where $L_{GL}=-logP(y|T;\theta )$ , $P(y|T;\theta )$ is defined in Eq. (5), $\lambda _{1}$ and $\lambda _{2}$ are hyper-parameters.

## Experiment

We use WIKIBIO^4^ and ROTOWIRE^9^ as benchmark datasets.

### Experiment on WIKIBIO

**Table 2:** Statistics of WIKIBIO dataset.

WIKIBIO	Tokens per reference	Infobox token per refe.	Tokens per infobox	Fields per infobox
Mean	26.1	9.5	53.1	19.7

**Table 3:** Dataset division of WIKIBIO.

WIKIBIO	Train	Valid	Test
Ratio (%)	80	10	10
Number	582660	72831	72831

#### Dataset and evaluation metrics

WIKIBIO contains 728,321 articles from the English version of Wikipedia. The first sentence of each article in WIKIBIO is extracted as the corresponding reference of the infobox. Table 2 shows the dataset statistics. There is an average of 26.1 tokens per reference, of which 9.5 tokens appear in the infobox. Each infobox has an average of 53.1 tokens and 19.7 fields. We divide the dataset into training (80%), validation (10%), and testing (10%) sets. The detail of dataset division is listed in Table 3. We use BLEU-4 and ROUGE-4 (F-measure) as automatic evaluation metrics. They are computed by NIST mteval-v13a.pl (BLEU) and MSR rouge-1.5.5 (ROUGE).

#### Implementation details

We use a transformer model, where the number of blocks is set to 6 and the number of heads is 8. The hidden units of are set to 512. The model dimensions in terms of word embeddings, position embeddings, and field embeddings are 452, 5, and 50, respectively.

We use an Adam optimizer^30^ and GELU activation function^31^ to train the model. For the hyper-parameters of Adam optimizer, the learning rate is initially set to 0.001. We half the learning rate when the model fails to improve performance on the validation sets in 2 epochs. The label smoothing factor is 0.05. We clip the gradients^32^, the maximum norm of the gradients is 5. In the inference state, we adapt nucleus sampling with p=0.95 and top-k sampling with k=30. Beam size is set to be 5. The maximum length of the generated sentence is limited to 150 by counting the length of the reference text. According to the experimental results on the validation set, the weight $\lambda _{1}$ of the table-to-text constraint loss is 0.2, and the weight $\lambda _{2}$ of the copy loss is 0.5.

#### Baselines

We compare our model with six baseline models. For each of them, we use the same parameter settings as the corresponding paper, and report the best experimental results of each baseline model, the baselines are as follows:

Table NLM^4^ is based on the conditional language model and introduces a copy mechanism to solve the problem of sparse data. The structured data are parsed both locally and globally, with a focus on the attribute information of words. Furthermore, Wikipedia’s biographical dataset WIKIBIO is created.

Order-plan model^5^ uses a content-based and link-based hybrid attention mechanism to plan the form of the content, and on the decoding side, an RNN network with a copy mechanism is used to solve the out-of-vocabulary problem.

Structure-aware Seq2seq model^1^ involves field gating and dual attention mechanisms. In the encoding phase, field information is integrated into the table representation by adopting field gating, and a dual attention mechanism consisting of word-level attention and field-level attention is proposed to effectively model the semantic information between the input tables and generated descriptions in the decoding phase.

FA+RL method^7^ uses an attention-based approach to encourage decoders to focus on uncovered attribute information and avoid missing critical attribute information; this is done using reinforcement learning to generate descriptions that are informative and faithful to table inputs.

NCP model^17^ is a two-stage model that combines content selection and content planning. First, a content plan is generated through the pointer generation network. Then, the content plan is employed as the input of the recurrent neural network to generate a description.

NCP+BAT^35^ is an end-to-end model that jointly learns the content planning and text generation. The content plan is integrated into the encoder-decoder model by using the coverage mechanism.

#### Overall experimental results

We carry out experiments on WIKIBIO dataset, and Table 4 shows the experimental results of the various models. To determine whether our model results in a statistical difference for the evaluation metrics, we utilize the paired T-Test in Table 4. It can be concluded from Table 4 that our model is different from the baseline models at a significant level of 0.01. We further compare the mean of our model and the baseline model. The mean values of our model are 46.87 and 42.28, respectively, which are higher than the mean values of all baseline models.

**Table 4:** BLEU and ROUGE Scores on the WIKIBIO Dataset. For each model, we report the ”mean standard deviation”. Compare with our model, $$*p<0.05$$, $$**p<0.01$$.

Model	BLEU		ROUGE	
Table NLM^4^	34.70	0.36**	25.80	0.36**
Order-plan^5^	43.91	0.35**	37.15	0.24**
Structure-aware^1^	44.89	0.33**	41.21	0.32**
FA+RL^7^	45.47	0.25**	41.54	0.35**
NCP^17^	43.12	0.32**	38.82	0.25**
NCP+BTA^35^	45.46	0.28**	40.31	0.27**
Ours	**46.87**	**0.33**	**42.28**	**0.29**
Transformer (base)	44.33	0.27	40.12	0.35
+ $L_{CL}$	45.93	0.34	41.36	0.33
+$L_{copy}$	45.28	0.41	41.09	0.23
+ top-p+top-k	44.79	0.34	40.57	0.28

**Figure 2:**
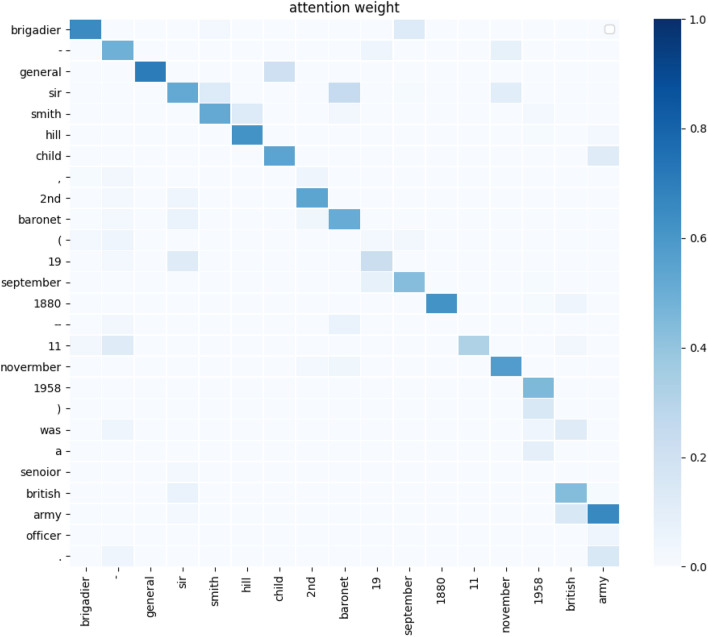
Weighted visualization results, with horizontal coordinates for the values in the table and vertical coordinates for the generated target text.

Transformer(base) represents a transformer-based data-to-text model without any learning task. Compared with the RNN-based Table NLM model, our Transformer model (base) uses the same input and search strategy, but the BLEU value and ROUGE value of our model are improved by 9.63 (from 34.70 to 44.33) and 14.32(from 25.80 to 40.12) , respectively. Thus, with sufficient data, the quality of text generation can be significantly improved by applying the transformer-based model. The NCP model, NCP+BAT model and FA+RL model improve the performance of the model by allowing the decoder to focus on key attribute information. Compared with the baseline models, our model is much better on ROUGE and BLEU. We apply table-to-text constraint loss (row 9) to enhance the representation of the table content, which makes the semantics of the table content and the target text closer. The BLEU value and ROUGE value of the model increased by 1.6 and 1.24 respectively. The experimental results confirm our theory that appropriate learning objectives can enhance the performance of the model. We adopt target text preprocessing and copy loss in the model (line 10) to faithfully copy the contents of the table, the BLEU value and ROUGE value reached 45.28 and 41.09, respectively. Compared with the Transformer(base) model, our model achieved 0.95 points higher on BLEU value and 0.97 points higher on ROUGE value. Experimental results show that $L_{CL}$ is more beneficial to text generation against the $L_{copy}$. In the eleventh row of Table 4, we use a combination of top-p and top-k sampling instead of beam search sampling, and this improves the BLEU score from 44.33 to 44.79 and the ROUGE score from 40.12 to 40.57. The new sampling policy alleviates problems such as redundancy and inconsistency and improves the quality of text generated.

We visualize the process of generating a paragraph description based on an infobox in Fig. 2 , where the horizontal coordinate represents the value in the table, and the vertical coordinate represents the generated text. The table word corresponding to the largest attention weight is selected as the word generated at the current moment. For example, when generating the third token, the word ”general” in the table receives the largest attention weight, so ”general” is used as the generated word. Most of the attention weights in Fig. 2 yield the desired results, further confirming the importance of our model.

**Table 5:** Table-to-text constraint loss effectiveness analysis on the WIKIBIO Dataset. “Speed” denotes the speed in the train phase. ”Param” represents trainable parameters number of the model (M is one million).

Model	BLEU	ROUGE	Sim(%)	Param	Speed(tokens/s)
Transformer(base)	44.33	40.12	15.9	63.8M	4847
+$L_{CL}$	45.93	41.36	25.8	63.8M	4736

**Table 6:** Results of the comparison between the reference text and generated text.

Field	Value	Text (R:Refrernce G:Generation)
Name	Marie Stephan	R: marie stephan , -lrb- born March 14, 1996 -rrb- is a professional squash player who represents france.
Birth date	14 March 1996	
Birth place	France	G: marie stephan , -lrb- born March 14, 1996 **in france** -rrb- is a professional squash player who represents france.
Residence	Nîmes: France	
Name	Darren m. swain	R: darren m.swain is an american politician, a democrat and a member of the maryland house of delegates.
Birth date	06 may 1970	
Birth place	North carolina	
Party	Democrat	G: darren m. swain **-lrb- born May 6, 1970 -rrb-** is an american democratic party and the member of the maryland house of delegates.
Order	Maryland house	
	Of delegates	
Name	Jim melrose	R: jim melrose -lrb- born 7 october 1958 -rrb- is a scottish retired professional footballer who played as a striker.
Birth date	7 october 1958	
Birth place	Glasgow: scotland	G: jim melrose -lrb- born 7 october 1958 **in glasgow** -rrb- is a former scottish footballer who played as a striker.
Position	Striker	
Honorific prefix	Brigadier-general	R: brigadier-general sir smith hill child, 2nd baronet, -lrb- 19 September 1880–11 November 1958 -rrb- was an officer in the british army **and a conservative party politician.**
Name	Sir smith hill child	
Honorific suffix	2nd baronet	
Birth date	19 september 1880	
Death data	11 november 1958	G: brigadier-general sir smith hill child, 2nd baronet -lrb- 19 september 1880–11 november 1958 -rrb- was a **senior** british army officer during the first world war.
Battles	First world war	
Branch	British army	

#### Table-to-text constraint loss effectiveness analysis

We study how the table-to-text constraint loss ($L_{CL}$) affects the similarity of source and target sentences. We adopt the cosine similarity^37^ to calculate the similarity between the source and the target sentences, where each sentence is represented by the mean value of word embedding, and the similarity calculation equation is defined as:

14 $$\begin{aligned} sim = cos({\hat{E}}_{enc},{\hat{E}}_{dec}) \end{aligned}$$

From the second to fourth columns of Table 5, it can be seen that the generation performance (BLEU and ROUGE) and sentence similarity (Sim) are higher than the transformer (base) by increasing the table-to-text constraint loss. This shows that there is a correlation between the performance of text generation and the similarity of sentences, the more similar the source and the target sentences, the better the performance of text generation. The experimental results prove that improving the similarity between the table and the target text is an effective method to improve the performance of the model.

We further analyze the efficiency of table-to-text constraint loss from the speed and performance of the model. Compared with the transformer (base), $L_{CL}$ achieves superior generation performance without any parameter increase. The BLEU value and ROUGE value are increased by 1.6 and 1.24 points, respectively, and the training speed is barely reduced approximately 1%. It shows that table-to-text constraint loss can improve the quality of text generation without sacrificing training speed.

#### Case study

Four of the generated texts are randomly selected for a comparison with the reference text, and the experimental results are shown in Table 6. More table-to-text generation examples are listed in Appendix A. ”Reference” indicates the reference text, and ”Generation” indicates the generated text. As seen in Table 6, there is redundancy in the first and third generated texts. Although the second generated text is not consistent with the reference text, the text generated by our model is more faithful to the table’s contents. In addition, our model can learn the relationship between ”north carolina” and ”American” without external knowledge. The last line generates text that does not fully describe the reference text, but the missing parts do not appear in the table. There are slight differences between generated text and reference text, but most of the generated text exactly replicates the content of the table, which is primarily due to our copy loss.

From the above analysis, it is clear that our model more reliably describes the table contents than other models, although there is a small amount of redundancy. Therefore, it is worth exploring whether the generated text should be closer to the reference text or more faithful to the table input.

### Experiment on ROTOWIRE

Experiments on the WIKIBIO dataset demonstrate the effectiveness of the model. In this part, we perform experiments on the ROTOWIRE dataset to prove the generality of the model. Compared with the WIKIBIO dataset, the ROTOWIRE dataset is basically in a digital format. Therefore, the model is required to understand the relationship between numerical data.

#### Dataset and evaluation metrics

ROTOWIRE dataset consists of (human-written) NBA basketball game summaries with their corresponding box-scores and line-scores. In the line-score tables, each team is described by 15 types of values. In the box-score tables, each player has 23 different types of values, each row corresponds to a player in the game. The average length of the summary is 337.1 tokens, and the vocabulary size is 11.3K. The summaries have been randomly split into training, validation, and test sets consisting of 3398, 727, and 728 summaries, respectively, the detail is shown in Table 7. We use BLEU-4 and several content-oriented metrics^9^ to evaluate model output. For content-oriented metrics, we apply the public IE system^17^ to extract relations. Content-oriented metrics include three aspects:

**Table 7:** ROTOWIRE dataset division.

ROTOWIRE	Train	Valid	Test
Ratio (%)	70	15	15
Number	3398	727	728

- Content Selection (CS) evaluates the recall rate and precision of extracted relations in the generated description and gold description.
- Relation Generation(RG) evaluates the number and precision of extracted relations in the generated description and input dataset.
- Content Ordering (CO) evaluates the normalized Damerau-Levenshtein Distance^34^ of the relations extracted in the generated description and the gold description.

#### Implementation details

We use a transformer model, where the number of blocks is set to 6, the number of heads is 8 and hidden units are 512. In the data preprocessing stage, the input table is converted into a fixed-length sequence of records. Each record consists of four types of information (entity, type, value and game information), the record embedding size is 128. Since there is no order relationship in the records, only learn the position embedding of the decoder in the transformer. Our model is trained with GELU activation function^31^ and Adam optimizer^30^. The learning rate is fixed to 0.0001 in the Adam optimizer. The label smoothing factor is 0.05. In the inference state, we adapt nucleus sampling with p=0.95 and top-k sampling with k=40. The maximum length of the generated sentence is limited to 600 by counting the length of the reference text. According to the experimental results on the validation set, the weight $\lambda _{1}$ of the table-to-text constraint loss is 0.2, and the weight $\lambda _{2}$ of the copy loss is 0.5.

**Table 8:** Automatic evaluation results on the ROTOWIRE validation set using BLEU, relation generation (RG) number $(\#)$ and precision (P%), content selection (CS) precision (P%) and recall (R%), content ordering(CO). - : unavailable experimental results.

Model	RG		CS		CO	BLEU
	#	P%	P%	R%	DLD%	
GOLD	23.32	94.77	100.00	100.00	100.00	100.00
Template^9^	**54.23**	**99.92**	26.60	**59.13**	14.39	8.62
CC^9^	23.95	75.10	28.11	35.86	15.33	14.57
NCP^17^	33.88	87.51	33.52	51.21	18.57	16.19
RCT^33^	32.11	91.84	35.59	48.98	20.70	16.42
Hierarch-k^36^	–	–	–	–	–	–
Ours	23.73	86.52	**40.69**	48.85	**23.72**	**19.52**

**Table 9:** Automatic evaluation results on the ROTOWIRE test set using BLEU, relation generation (RG) number $(\#)$ and precision (P%), content selection (CS) precision (P%) and recall (R%), content ordering(CO). - : unavailable experimental results.

Model	RG		CS		CO	BLEU
	#	P%	P%	R%	DLD%	
GOLD	24.14	94.89	100.00	100.00	100.00	100.00
Template^9^	**54.21**	**99.94**	27.02	**58.22**	15.07	8.58
CC^9^	23.72	74.80	29.49	36.18	15.42	14.19
NCP^17^	34.28	87.47	34.18	51.22	18.58	16.50
RCT^33^	31.47	91.46	36.09	48.01	20.86	16.85
Hierarch-k^36^	21.17	89.46	39.47	51.64	18.90	17.50
Ours	24.12	87.05	**40.43**	48.42	**23.19**	**19.43**

#### Experimental results

On the ROTOWIRE dataset, we use five baseline models. For each of them, we adopt the best experimental results in each paper. GOLD represents the experimental results on the gold summary. The baseline models are as follows:

CC^9^ adopts a conditional copy mechanism in the encoder-decoder model. Template is a template-based generator model same as the one used in^9^ which generates 8 templated sentences from the training set: a sentence about the teams playing in the game, 6 highest-scoring players sentences and a conclusion sentence. The NCP model^17^ combines content selection and content planning in a neural network architecture. The RCT model^33^ considers the row, column, and time dimension information in the input table, and then combines the three-dimensional representations into a dense vector through the table cell fusion gate. The Hierarch-k model^36^ employ a novel two-level Transformer encoder to hierarchically capture the structure of the data. Two variants of hierarchical attention mechanism are used to get context as the input of decoder.

Table 8 displays the automatic evaluation results of the ROTOWIRE dataset on the validation set. Our model achieves significantly higher results than all other baseline models in BLEU, CS precision and CO metrics. Our model generates almost the same number of records as the baseline model CC, but has a significant improvement in other metrics. Comparing to CC model, our model is 12.58% higher on CS precision, 12.99% higher on CS recall, 11.42% higher on RG precision, 8.39% higher on CO metric, and 4.95 points higher on BLEU. Comparing to NCP and RCT, our model is better on CS precision, Content Ordering metric and BLEU. This may be due to the fact that our model generates almost the same number of relations as the gold summary, reducing the normalized DL Distance^34^ between the two sequences of relations. However, our model performs lower RG precision and lower CS recall with the number of relations decreases. Experimental results on the test set are shown in Table 9. As can be seen from Table 8 and Table 9, the experimental results of the test set and the validation set are not significantly different. Compared with all other contrast models, our model gets higher CS precision, CO metric and BLEU. Our model yields a more outstanding BLEU value (19.43 vs. 17.50) against the best baseline Hierarch-k. This shows that the text generated by our model is closer to the gold summary and can generate more fluent target text.

#### Ablation studies

Next, we conduct ablation studies to evaluate the various components of our model. This is:

- The table-text constraint loss to constrain the complex structure of the table by the target text.
- The copy loss aiming at providing accurate guidance to the pointer-generator network.
- The search strategy to reduce the probability of problems such as sentence repetition and boring.

##### Removing the table-text constraint loss

In this configuration, we employ the same search strategy and copy loss as our model, but the model is trained without table-text constraint loss. It can be concluded from Table 10 (-TT_CL) that almost the same number of records as our model have been extracted, but the accuracy is decreased by 6.93%. CS precision and CS recall are dropped by 4.25% and 4.34%, respectively.

##### Removing the copy loss

At this stage, the copy loss is removed from our model. From the results in Table 10 (-COPY), we can conclude that all evaluation results are degraded. However, the BLEU value did not change significantly. We can accurately copy words from tables by copy loss task.

##### Changing the search strategy

In this part, we keep the table-text constraint loss and copy loss of our model, replace our search strategy with beam search, and set the beam size to 4. Table 10 (-DE_STR) shows that, changing the search strategy, CS precision and CS recall are degraded by 2.45% and 1.89%, respectively, the content ordering metrics is degraded by 1.36%, and the RG precision is dropped by 3.02%. Experimental results show that the combination of top-p and top-k search strategies can improve the performance of the model.

**Table 10:** Ablation results on the ROTOWIRE test set.

Model	RG		CS		CO	BLEU
	#	P%	P%	R%	DLD%	
Ours	24.12	**87.05**	**40.43**	**48.42**	**23.19**	**19.43**
-TT_CL	24.20	80.12	36.18	44.08	21.58	18.98
-COPY	21.45	82.17	38.09	47.17	22.67	19.18
-DE_STR	**25.89**	84.03	37.98	46.53	21.83	18.76

## Conclusions

In this paper, we propose a novel transformer-based table-to-text generation algorithm. We first apply the table-text constraint loss operation to effectively learn the semantic representation of the table. Then, we propose a copy loss with target text processing to gain the precise positions in which we should copy. Finally, a combination of the top-p and top-k search strategies is adopted to improve the text generation quality of the model. Experiments are conducted on two datasets, Biography, and basketball game, in different domains. Our final model achieves state-of-the-art performance on BLEU, ROUGE and Content-oriented metics. We also use a paired T-Test to verify that our model is significantly different from other models. In addition, we conduct ablation experiments to further verify the effectiveness of the various components of our algorithm.In the future, we will research algorithms for optimizing multi-task learning weights, and use learnable weights instead of fixed weights.

## Supplementary Information

Supplementary Information.

## Data Availability

All data included in this study are available upon request by contact with the corresponding author.
