# Supplementary material for: Table to text generation with accurate content copying
Source: Sci Rep. 2021 Nov 23;11:22750. doi: 10.1038/s41598-021-00813-6 (PMC8611016; doi:10.1038/s41598-021-00813-6)
Supplement: Supplementary file 1 — Supplementary Information. [file 41598_2021_813_MOESM1_ESM.pdf]

Appendix A. Table-to-text Generation Examples

More examples of generated text are shown in Table 1. Our algorithm generates coherent texts that are faithful to the tabular data in Table 1.

Table 1. Results of the comparison between the reference text and generated text

<table>
<tr><th>Field</th><th>Value</th><th>Text (R:Refrernce G:Generation)</th></tr>
<tr><td>Name</td><td>bus griffiths</td><td rowspan="8">R: gilbert joseph “ bus ” griffiths -lrb- 1913 – september 25 , 2006 -rrb- was a cartoonist , lumberjack , and fisherman .

G: gilbert joseph griffiths -lrb- 1913 – september 25 , 2006 -rrb- was a canadian cartoonist .</td></tr>
<tr><td>Birth name</td><td>gilbert joseph griffiths</td></tr>
<tr><td>Birth date</td><td>1913</td></tr>
<tr><td>Birth place</td><td>moose jaw; saskatchewan; canada</td></tr>
<tr><td>Death date</td><td>september 25, 2006</td></tr>
<tr><td>Death place</td><td>comox, british columbia, canada</td></tr>
<tr><td>Nationality</td><td>canadian</td></tr>
<tr><td>Cartoonist</td><td>y</td></tr>
<tr><td>Name</td><td>binky jones</td><td rowspan="12">R: john joseph “ binky ” jones -lrb- july 11 , 1899 in st. louis , missouri – may 13 , 1961 -rrb- , was a professional baseball player who played shortstop for the brooklyn robins in ten games during the 1924 season .

G: binky jones -lrb- july 11 , 1899 – may 13 , 1961 in st. louis , missouri -rrb-, was a former professional baseball player who played shortstop for the brooklyn robins from april 15 to april 27 robins in the 1924 .</td></tr>
<tr><td>Position</td><td>shortstop</td></tr>
<tr><td>Birth date</td><td>11 july 1899</td></tr>
<tr><td>Birth place</td><td>st.louis, missouri</td></tr>
<tr><td>Death date</td><td>13 may 1961</td></tr>
<tr><td>Death place</td><td>st.louis, missouri</td></tr>
<tr><td>Debutdate</td><td>april 15</td></tr>
<tr><td>Debutyear</td><td>1924</td></tr>
<tr><td>Debutteam</td><td>brooklyn robins</td></tr>
<tr><td>Finaldate</td><td>april 27</td></tr>
<tr><td>Finalyear</td><td>1924</td></tr>
<tr><td>Finalteam</td><td>brooklyn robins</td></tr>
<tr><td>Name</td><td>arthur barr-kemp</td><td rowspan="9">R: arthur barr-kemp -lrb- 6 september 1921 – 25 september 2003 -rrb- was an australian rules footballer who played in the vfl from 1943 to 1946 for the richmond football club .

G: arthur barr-kemp -lrb- 6 september 1921 – 25 september 2003 -rrb- was an australian rules footballer who played for the richmond club from 1943 to 1946 .</td></tr>
<tr><td>fullname</td><td>arthur lionel barr-kemp</td></tr>
<tr><td>Birth date</td><td>6 september 1921</td></tr>
<tr><td>Death date</td><td>25 september 2003</td></tr>
<tr><td>Originalteam</td><td>brunswick</td></tr>
<tr><td>Years</td><td>1943-1946</td></tr>
<tr><td>Clubs</td><td>richmond</td></tr>
<tr><td>Statsend</td><td>1946</td></tr>
<tr><td>Article title</td><td>arthur barr-kemp</td></tr>
</table>
